# Levofloxacin induced Stevens-Johnson syndrome/ toxic epidermal necrolysis overlap syndrome: case reports

**DOI:** 10.1186/2045-7022-4-S3-P91

**Published:** 2014-07-18

**Authors:** Maria Gloria Aversano, Jan Schroeder, Antonella Citterio, Joseph Scibilia, Chiara Gamba, Luca Giuseppe Balossi, Ambra Mascheri, Laura Michelina Losappio, Elide Anna Pastorello

**Affiliations:** 1 Niguarda Ca' Granda Hospital, Allergology and Immunology Unit, Italy; 2 Niguarda Ca' Granda Hospital, Burn/ Intensive Care Department, Italy; 3 Papa Giovanni XXIII Hospital, GISED Research Center, Italy

## 

Fluoroquinolones are widely used because of their broad spectrum of activity. Their benefit-risk profile needs careful evaluation as they can induce T cell-dependent reactions including Stevens-Johnson syndrome (SJS) and toxic epidermal necrolysis (TEN). Here we present two rare cases of SJS/TEN overlap syndrome and SJS caused by levofloxacin. Case 1. A 70-year-old woman with a history of chronic obstructive pulmonary disease was treated with levofloxacin for acute bronchitis. After taking medications for 10 days she complained of generalized skin eruptions and mucosal erosions. On the day of admission in the burn intensive care department 28 % of her body surface was covered by rash, blistering and epidermal peeling; 65 % of body surface presented dusky red skin. Levofloxacin was stopped and our patient was treated with topical solution to keep the wounds moist, fluid replacement, well balanced nutrition, systemic corticosteroids and intravenous immune globulin (IVIG). We also administrated teicoplanin because of S. aureus infection of central i.v. catheter confirmed by a positive culture. She was subsequently treated with meropenem because of a Klebsiella pneumoniae urinary tract infection. The main laboratory findings were: anemia, leukocytosis and increased CRP levels. The diagnosis was performed on the basis of clinical and histological features. Gradually her symptoms improved and the epidermal peeling also subsided. Case 2. A 72-year-old woman was referred to our department with multiple skin lesions involving more than 50 % of the total body surface area occurred after 2 weeks of starting levofloxacin treatment for pneumonia. She developed high fever and had the appearance of a severe burn patient. Laboratory analysis showed anaemia, leukocytosis, hypoalbuminaemia and elevated procalcitonin and CRP. The levofloxacin treatment was stopped immediately and replaced with teicoplanin and meropenem. Subsequently because of A. baumanii complex infection of the central i.v. catheter she was treated also with colistin. Our management included IVIG treatment, intravenous glucocorticoids, proper fluid balance, attention to nutritional status and pain relief. The skin lesions healed within 3 weeks and the patient left our department in good general condition.

## Conclusions

Even if there are little published data on levofloxacin TEN/SJS, this fluoroquinolone can be implicated in these delayed ADR requiring early diagnosis and careful monitoring.

